# Repurposing of the Drug Tezosentan for Cancer Therapy

**DOI:** 10.3390/cimb45060325

**Published:** 2023-06-11

**Authors:** Eduarda Ribeiro, Nuno Vale

**Affiliations:** 1 OncoPharma Research Group, Center for Health Technology and Services Research (CINTESIS), Rua Dr. Plácido da Costa, 4200-450 Porto, Portugal; eduardaprr@gmail.com; 2 CINTESIS@RISE, Faculty of Medicine, University of Porto, Alameda Professor Hernâni Monteiro, 4200-319 Porto, Portugal; 3 Institute of Biomedical Sciences Abel Salazar (ICBAS), University of Porto, Rua de Jorge Viterbo Ferreira 228, 4050-313 Porto, Portugal; 4 Department of Community Medicine, Health Information and Decision (MEDCIDS), Faculty of Medicine, University of Porto, Rua Doutor Plácido da Costa, 4200-450 Porto, Portugal

**Keywords:** tezosentan, cancer, endothelin receptors, drug repurposing

## Abstract

Tezosentan is a vasodilator drug that was originally developed to treat pulmonary arterial hypertension. It acts by inhibiting endothelin (ET) receptors, which are overexpressed in many types of cancer cells. Endothelin-1 (ET1) is a substance produced by the body that causes blood vessels to narrow. Tezosentan has affinity for both ET_A_ and ET_B_ receptors. By blocking the effects of ET1, tezosentan can help to dilate blood vessels, improve the blood flow, and reduce the workload on the heart. Tezosentan has been found to have anticancer properties due to its ability to target the ET receptors, which are involved in promoting cellular processes such as proliferation, survival, neovascularization, immune cell response, and drug resistance. This review intends to demonstrate the potential of this drug in the field of oncology. Drug repurposing can be an excellent way to improve the known profiles of first-line drugs and to solve several resistance problems of these same antineoplastic drugs.

## 1. Introduction

Nowadays, cancer remains a significant health problem worldwide. In 2020 there were an estimated 19.3 million new cases of cancer and almost 10 million deaths from cancer [1]. Despite huge improvements, current anticancer pharmacological therapies are effective in a limited number of cancer cases. Tumors with a high mortality rate, targets not reachable by chemotherapy, and chemotherapy resistance, represent the current challenges of cancer treatments [2]. As the pharmaceutical productivity and drug efficacy in oncology seem to have reached a plateau, ‘drug repurposing’—meaning the use of old drugs, already in clinical use, for a different therapeutic indication—is a promising and viable strategy to improve cancer therapy. Opportunities for drug repurposing are often based on occasional observations or on time-consuming pre-clinical drug screenings that are often not hypothesis-driven [3]. This approach is greatly beneficial because of the main benefits such as the reduced development timelines with an average saving of 5–7 years, high approval rates, lower development costs, and, as they are already approved drugs, these compounds have already been tested in humans, so comprehensive information exists on their pharmacology, dose, possible toxicity, and formulation [4,5]. A possible solution to tackle the complexity of treating different types of cancer is to focus on how a specific treatment functions at a molecular level. By understanding this, we can identify which types of cancer are more likely to respond to that treatment [4,6]. However, it is essential to note that the effects of drugs on cancer cells vary depending on the specific drug and cancer type being examined. This is important to consider, as the results observed in laboratory studies may not always translate to the same effects in humans.

One such approach is the use of vasodilators, drugs originally development to use in the management of hypertension, angina, preeclampsia, stroke, heart failure, chronic kidney disease, and myocardial infarction. Examples of vasodilators include drugs such as angiotensin-converting enzyme inhibitors, angiotensin receptor blockers, calcium channel blockers, nitrates, PDE5 inhibitors, and endothelin antagonists, among others. These drugs work through different mechanisms to relax the smooth muscles in blood vessels, thereby reducing blood pressure and improving blood flow throughout the body [7].

Tezosentan, a vasodilator drug originally developed to treat pulmonary arterial hypertension acts through inhibiting endothelin (ET) receptors, which are overexpressed in many types of cancer cells [8].

Therefore, additional research is necessary to fully comprehend the impact of tezosentan on cancer cells and determine their potential as a cancer treatment. In the following section, we review the current understanding of the mechanisms of action of tezosentan in cancer and its clinical applications as a novel anticancer agent.

## 2. Structure of Tezosentan and Mechanism of Action

**Figure 1:**
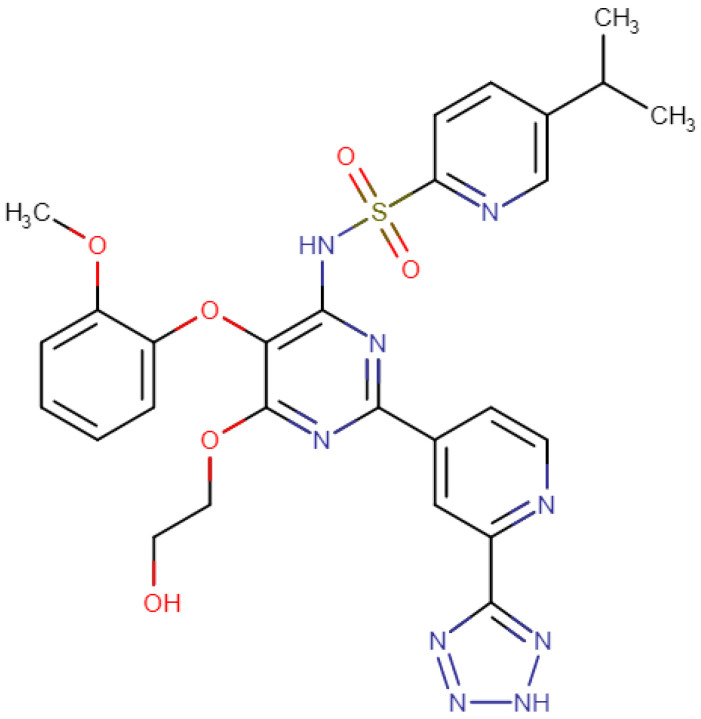
Chemical structure of the drug tezosentan (figure edited from DrugBank.com; accessed on 25 March 2023).

Tezosentan is a small molecule that belongs to a class of drugs known as ET receptor antagonists, which work through blocking the effects of a hormone called endothelin. The chemical name of tezosentan is N-[(2R)-6-(2,6-dimethylphenyl)-5-[(3R)-3-methyl-2,3,4,5-tetrahydro-1,5-benzoxazepin-5-yl]pyridazin-3-yl]-4-methoxybenzamide, and its chemical formula is C_30_H_31_N_5_O_2_. Tezosentan belongs to the class of organic compounds known as pyridinylpyrimidines and is composed of three primary structural moieties (Figure 1), namely a pyridazine ring, a tetrahydrobenzoxazine ring, and a phenyl group. These moieties are connected through a linker, with the pyridazine ring being linked to the tetrahydrobenzoxazine ring and the phenyl group being attached to the pyridazine ring. Additionally, the phenyl group of the molecule is substituted with a methoxy group (-OCH_3_).

Endothelin-1 (ET1) is a naturally occurring substance that is produced by the body and has potent vasoconstrictive effects, meaning it causes blood vessels to narrow. ET1 is produced by the endothelial cells that line blood vessels, and acts on two types of receptors: endothelin type A (ET_A_) and endothelin type B (ET_B_). Tezosentan has affinity for both ET_A_ and ET_B_ receptors [9,10]. By blocking the effects of ET1, tezosentan can help to dilate blood vessels, improve the blood flow, and reduce the workload on the heart. This can help to alleviate symptoms of heart failure and improve overall cardiac function. The ET_A_ receptor is primarily found in vascular smooth muscle cells and is responsible for causing blood vessels to narrow and for promoting cell growth. The ET_B_ receptor is found in various tissues, including the brain, blood vessels, and heart, and can have different effects depending on where it is located. In blood vessels, it can cause both constriction and dilation, while in cardiac fibroblasts it is believed to play a role in fibrosis, which is the buildup of excess connective tissue that can impair heart function [11].

Tezosentan is rapidly distributed after intravenous administration, with a volume of distribution of approximately 3 L/kg. The drug has a linear pharmacokinetic profile over a wide range of doses. Tezosentan is metabolized primarily by the liver via the cytochrome P450 3A4 enzyme system, and its clearance is primarily via the biliary route. The drug has a half-life of approximately 30 min and is eliminated from the body within 24 h after administration [9,12].

## 3. Clinical Use against Cancer

### 3.1. Endothelin-1 Receptor

**Figure 2:**
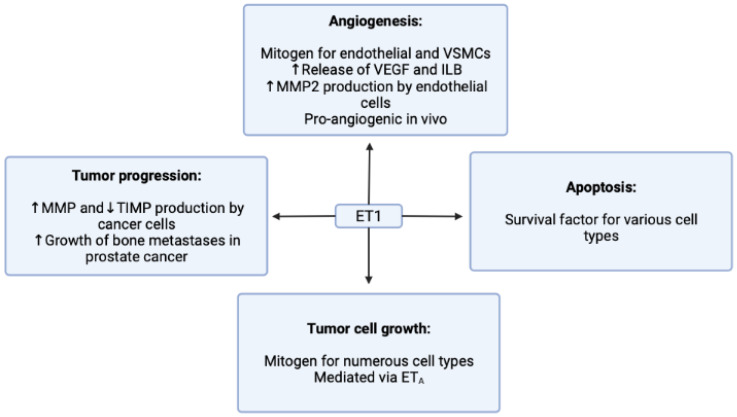
Actions of endothelin-1 in cancer.

The ET1 signaling pathway can promote various cellular processes such as proliferation, survival, epithelial-to-mesenchymal transition, neovascularization, immune cell response, and drug resistance, but the effects may vary depending on the context (Figure 2) [13]. Several tumor cell lines have demonstrated increased levels of ET1 production, highlighting the targeting of endothelin receptors as an important approach for cancer therapy [14,15,16,17,18].

**Figure 3:**
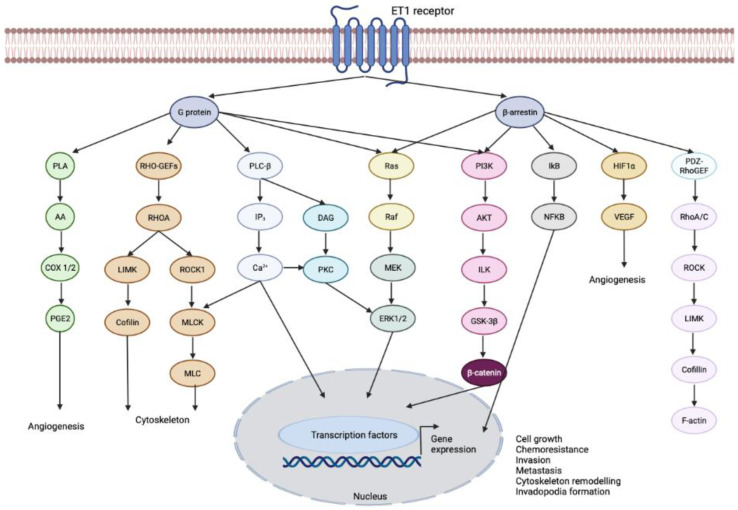
The ET1 signaling network. ET1, a signaling molecule involved in cancer, activates several pathways that contribute to various cellular processes. Upon binding to its receptor, it initiates a cascade of events involving G-protein coupled receptor activation and the activation of primary effectors. One of the pathways activated by ET1 is through the activation of PLCβ. This enzyme cleaves a molecule called phosphatidylinositol-4,5-bisphosphate (PtdIns(4,5)P2) into diacylglycerol (DAG) and inositol triphosphate (IP3). This leads to an increase in calcium levels and the activation of protein kinase C (PKC). Additionally, this pathway triggers the activation of members of the MAPK family, including ERK1/2, which are important for cellular signaling. ET1 activation also stimulates the Ras/Raf/MEK pathway, which converges on the activation of ERK1/2. This pathway plays a role in transmitting signals that regulate cell growth and proliferation. Furthermore, ET1 stimulation activates phospholipase A (PLA), resulting in the release of arachidonic acid (AA) and the activation of cyclooxygenase-1 (COX-1) and COX-2. These enzymes are involved in the production of prostaglandin E2 (PGE2). ET1 also activates PI3K, leading to the activation of AKT, integrin-linked kinase (ILK), and glycogen synthase kinase (GSK)-3β, which in turn stabilizes β-catenin. Importantly, ET1 can also activate ERK1/2 and PI3K/AKT/β-catenin signaling through the involvement of β-arrestin1. Through β-arrestin1, ET1 also activates nuclear factor-kB (NF-kB) signaling by inhibiting NF-kB inhibitor (IkB), leading to the dissociation and subsequent nuclear localization of active NF-kB. Additionally, ET1 activates PDZ-RhoGEF, leading to the activation of Rho-A and -C GTPases. This activation triggers Rho-dependent signaling events through RHO-associated coiled-coil containing protein kinase 1 (ROCK1), resulting in the activation of LIMK and the inhibition of cofilin. Collectively, these signaling pathways, orchestrated by ET-1R, promote cell growth, chemoresistance, angiogenesis, cytoskeleton remodeling, invadopodia formation, and metastasis. By understanding and targeting these pathways, it may be possible to develop therapeutic strategies to intervene in cancer progression.

The ET_B_ signaling pathway has been found to promote the migration and proliferation of microvascular endothelial cells [19]. ET_A_ signaling, on the other hand, induces angiogenesis, particularly through the induction of vascular endothelial growth factor [20,21]. ET1 triggers the activation of phospholipase C β (PLCβ), which increases intracellular calcium ion levels and activates protein kinase C. It also activates phosphoinositide 3-kinase (PI3K), c-Jun N-terminal kinase (JNK), extracellular-signal-regulated kinase/mitogen-activated protein kinase (ERK/MAPK), and epidermal growth factor receptor (EGFR) signaling (Figure 3). Additionally, ET1-induced signaling leads to the activation of FAK and paxillin. Blocking ET_A_ and ET_B_ receptors has shown promise in treating systemic pulmonary hypertension and chronic heart failure by inhibiting endothelial cell proliferation and survival [22]. In cancer therapy, inhibition of endothelin receptor signaling has shown potential.

Changes in the expression of ET1 and its associated receptors and signaling pathways can disrupt normal cellular processes and contribute to the development and progression of tumors. This can occur through both autocrine signaling (when cells produce ET1 to stimulate their own growth) and paracrine signaling (when cells produce ET1 to stimulate the growth of neighboring cells). These changes may be caused by genetic mutations or epigenetic modifications, which can lead to aberrant expression of ET1 and alterations in its downstream signaling pathways [13].

Due to the significant involvement of ET_A_ in the biology of tumor cells, there has been greater emphasis on the development of selective antagonists targeting ET_A_ compared to those targeting ET_B_ in the treatment of cancer. This is reflected in Table 1, which demonstrates a higher number of ET_A_-selective antagonists developed for this purpose.

Based on safety data on other ET1 receptor antagonists, bosentan has the ability to affect specific enzymes in the body, including CYP2C9, CYP2C19, and CYP3A4. These enzymes play a role in the breakdown of many contraceptives, which means that when bosentan is taken together with these contraceptives, there is a possibility of reduced effectiveness. Patients with moderate to severe liver problems should exercise caution when using bosentan because it can lead to increased levels of liver amino transferase. Additionally, bosentan has been found to have the potential to cause birth defects. Ambrisentan, when used in combination with drugs such as ciclosporin, ketoconazole, or omeprazole, which affect the activity of CYP enzymes, can potentially result in drug interactions. Similar caution is recommended for inhibitors or inducers of P-glycoprotein (P-gp), UDP-glucuronosyltransferase, and organic-anion-transporting polypeptide (OATP) when co-administered with ambrisentan. Ambrisentan is not recommended for patients with severe liver impairment due to the involvement of the liver and bile in its metabolism and elimination. Macitentan is not recommended for use in pregnant women due to the risk of causing birth defects, and in individuals with severe liver dysfunction or elevated liver enzyme levels [23]. Tezosentan should be avoided in patients with clinically significant renal failure [10]. With this, tezosentan seems to be the safest ET1 receptor inhibitor to be tested as an antineoplastic drug.

### 3.2. Endothelin Receptor Type A

Several types of cancer, including colorectal, ovarian, and prostate tumors, have been shown to have increased expression of ET_A_ receptors in malignant tissue, as demonstrated through immunohistochemistry and autoradiography [24,25,26]. In fact, ET_A_ receptor expression levels in prostate tumors have been found to correlate with both the Gleason score and the presence of metastasis [27].

Akhavan et al., found that blocking ET1 receptors with a selective ET_A_ receptor antagonist, atrasentan, inhibited the growth of prostate cancer cells in vitro and in vivo by inducing apoptosis and reducing angiogenesis [28]. The results of another study on LIM1215 and HT29 colorectal cancer cell lines have shown that ET1 can promote cell growth through the ET_A_ receptor. However, it is not yet clear whether this effect is due to a mitogenic stimulus, an antiapoptotic signal, or a combination of both. Nevertheless, the findings of previous studies that have shown that ET1 is produced by colorectal cancers, along with the results of this study, suggest that ET1 may act as a mitogen in colorectal cancer [29,30]. Therefore, targeting the ET_A_ receptor with antagonists may have therapeutic potential for the treatment of colorectal cancer.

There is some debate over the role of ET1, which acts as both an autocrine and paracrine cytokine. However, findings of Liu et al., on gastric cancer cell lines suggest that activating the ET1/ET_A_ pathway contributes to cell proliferation, migration, and antiapoptosis. By acting mainly through ET_A_, ET1 plays a crucial role in promoting the development of gastric cancer, indicating that inhibiting the ET1/ET_A_ axis could potentially improve treatment outcomes [31]. Another study demonstrates that the drug ambrisentan, another ET_A_ receptor antagonist, inhibits in vitro cancer cell migration and invasion in COLO-357 metastatic pancreatic adenocarcinoma, OvCar3 ovarian carcinoma, MDA-MB-231 breast adenocarcinoma, and HL-60 promyelocytic leukemia. In vivo this drug has demonstrated its potential in reducing metastasis of a metastatic breast cancer into the lung and liver, thereby decreasing mortality [32]. Atrasentan (ABT-627), a specific antagonist of the endothelin-A receptor, has shown promising results in treating men with hormone-refractory metastatic prostate cancer. The safety profile of this drug aligns with its pharmacologic activity and makes it suitable for long-term, noncytolytic therapy. Further research is necessary to investigate its efficacy in earlier stages of prostate cancer and in other types of cancer in which the endothelin pathway may be involved [33].

Arabanian et al., demonstrate that ET_A_ is upregulated in leukemic cells that express high levels of *Hoxa9* and *Meis1*. Overexpression of ET_A_ has been shown to promote cell proliferation and enhance the repopulating capacity of *Hoxa9*-expressing cells. Moreover, cancer progression and metastasis rely heavily on the resistance to apoptosis, and leukemia-inducing cells require close contact with their niche to survive. This contact is supported and maintained by various surface molecules that modulate the homing capacity of leukemic cells and their ability to proliferate and remain quiescent [34]. Identification of these surface molecules can be helpful in targeting the residual leukemia-inducing cells after therapy. In this study, they investigated whether ET_A_ signaling contributes to protection against drug-induced apoptosis in leukemia cells [35]. They used daunorubicin, a common chemotherapeutic agent used in leukemia treatment, to induce apoptosis in the cells. The results suggest that upregulation of ET_A_ may contribute to chemo-resistance in leukemic cells in response to daunorubicin. ET_A_ may function as a signaling molecule that promotes cell growth and induces protection against cytotoxic effects. In conclusion, targeting ET_A_, along with other chemotherapies, may have therapeutic benefits for leukemia by overcoming resistance to drug-induced apoptosis. ET_A_ inhibitors could potentially be used in the treatment of leukemia to enhance the effectiveness of chemotherapy. Further studies are needed to investigate the mechanism of ET_A_ function using additional chemotherapeutic agents and ET_A_ knockdown models as controls [36].

#### ATP-Binding Cassette Subfamily B Member 1 (ABCB1) Protein

Upregulation of transmembrane transporters such as ATP-binding cassette subfamily B member 1 (ABCB1 or P-glycoprotein) in tumor cells contributes to drug resistance. The ABCB1 protein is known for its involvement in multidrug resistance of tumor cells by preventing intracellular accumulation of cytotoxic drugs. It serves as a membrane efflux that extrudes the drugs from the cancer cells, reducing their effectiveness [37,38]. This is a major problem in cancer treatment, as drug resistance can limit the effectiveness of chemotherapy and ultimately lead to treatment failure.

Several studies have shown that ABCB1 overexpression is associated with poor prognosis in various types of cancer, including breast [39], lung [40], colon [41], and ovarian cancer [42,43,44,45]. In addition, ABCB1 inhibitors have been investigated as potential drugs to overcome drug resistance in cancer cells. In that study, Englinger *et al*., found that a specific signaling pathway, called the ET1/ET_A_ pathway, was overactive in a type of lung cancer cell called DMS114/NIN, and this contributed to the cells’ resistance to a cancer drug called nintedanib. The overactive pathway led to increased levels of certain proteins (ABCB1, PKC, and NFκB), which are known to be involved in drug resistance. The researchers tested tezosentan, which blocks the ET_A_ protein, and found that it decreased the levels of ABCB1, PKC, and NFκB proteins in the cells. Importantly, this also led to a significant increase in the cells’ sensitivity to nintedanib. In summary, blocking the ET_A_ pathway using tezosentan could be a promising strategy to overcome resistance to nintedanib in cancer cells [46].

### 3.3. Endothelin Receptor Type B

ET_B_ is overexpressed in bladder carcinoma [47], melanoma [48], small-cell lung cancer [49], vulvar cancer [50], clear-cell renal cell carcinoma [51], esophageal squamous cell carcinoma [52], and glioblastoma [53].

Methylation of the ET_B_ gene has also been found to be relatively increased in several cancer cell lines from the prostate, bladder, and colon, with a corresponding downregulation of transcription [54]. This suggests a potential mechanism for the reduced expression of ET_B_ receptors in malignant tissue.

One of the main signaling pathways activated by ET_B_ in cancer is the PI3K/AKT pathway, which is involved in cell growth and survival [55,56]. ET_B_ can also activate the MAPK/ERK pathway, which is involved in cell proliferation and differentiation [57]. In addition, ET_B_ can stimulate the expression of various genes that promote cancer cell invasion and metastasis [58].

Melanocytes use a surface receptor called ET_B_ to produce and transport melanin, and it has been found that this receptor works in synergy with many other factors involved in the melanin pathway [59]. However, studies have shown that using ET_B_ antagonists can slow the growth of melanoma cells and increase their programmed cell death, or apoptosis [60]. Research has also indicated that ET_B_ antagonists can prevent the progression of melanoma induced by ET and stabilize metastatic disease, making it an effective clinical approach for treating melanoma [61,62].

A study led by Lahav et al., indicates that the ET_B_ inhibitor BQ788 is a potent agent for inhibiting human melanoma tumor growth in a nude mouse model, with some tumors being completely halted. Although the underlying mechanism is not yet fully understood, experiments conducted in culture suggest that BQ788 may slow melanoma cell growth, induce differentiation, and ultimately lead to cell death. For example, some lines showed a slower growth rate, increased pigmentation, and flattened appearance, while others displayed dendritic morphology characteristic of differentiated melanocytes. The most sensitive cell lines showed a loss of viability accompanied by cytoplasmic vacuoles and increased TUNEL staining, indicative of apoptosis. Although further investigation is needed to determine whether BQ788 is the most effective ET_B_ antagonist for stopping melanoma growth in vivo, there is evidence that such drugs can be well tolerated. Future experiments should explore why systemic treatment with BQ788 results in variable responses in different animals, and whether the effects are reversible. It will also be important to determine whether ET_B_ antagonists can inhibit metastasis in addition to tumor growth [63].

Another study on melanoma has identified ET_B_, which is associated with aggressive phenotypes, including the ability of melanoma cells to mimic blood vessels. By screening a variety of benign and malignant pigment cell lesions using immunohistochemistry and quantitative reverse transcription PCR analysis, the authors have found that ET_B_ is a marker of tumor progression in malignant melanoma and could be a clinically relevant target for the development of small molecules that can block ET. Furthermore, an orally active ET_B_ antagonist called A-192621 has displayed antitumor activity against established melanoma expressing ET_B_, making it a promising candidate for targeted therapy. ET-1 and ET-3 are molecules that trigger several molecular effectors involved in melanoma progression, including cell–cell adhesion and communication molecules, tumor proteases, and integrins, through the activity of ET_B_. By blocking this receptor with small molecules, melanoma growth can be inhibited, providing new possibilities for integrated treatments for this malignancy. This knowledge is particularly important given the known resistance of melanoma to current therapies [64].

Because of its role in promoting cancer cell growth and survival, ET_B_ has become a potential target for cancer therapy. The drugs that target ET_B_ work through blocking the activity of ET_B_ and inhibiting its downstream signaling pathways, thereby reducing cancer cell growth and invasion.

#### Nitric Oxide (NO) Release

When endothelin-1 binds to ETB receptors on endothelial cells, it triggers the synthesis of nitric oxide (NO) and the subsequent relaxation of smooth muscle [65]. Nitric oxide (NO), also known as nitrogen monoxide, is a naturally occurring gas that is soluble in water and acts as a free radical. Its presence is critical in several physiological and pathological processes. NO is produced by the oxidation of l-arginine through the catalytic activity of NO synthases (NOSs). This process requires nicotinamide adenine dinucleotide phosphate (NADPH) and O_2_ as cosubstrates, and it results in the production of NO and l-citrulline as byproducts. NO’s role in many bodily functions occurs primarily through a cyclic guanosine monophosphate (cGMP)-dependent pathway, leading to vasodilation, neurotransmission, inhibition of platelet aggregation, and smooth muscle relaxation. Another pathway for NO activity is cGMP-independent, and it involves the reaction of NO with molecular oxygen, superoxide (O2-), thiols, and transition metals such as zinc. Additionally, NO can directly modify proteins without enzyme involvement through nitration or nitrosylation. S-nitrosylation, a reversible modification, involves the signaling of cysteine thiol residues and regulates the function of several intracellular proteins [66].

**Figure 4:**
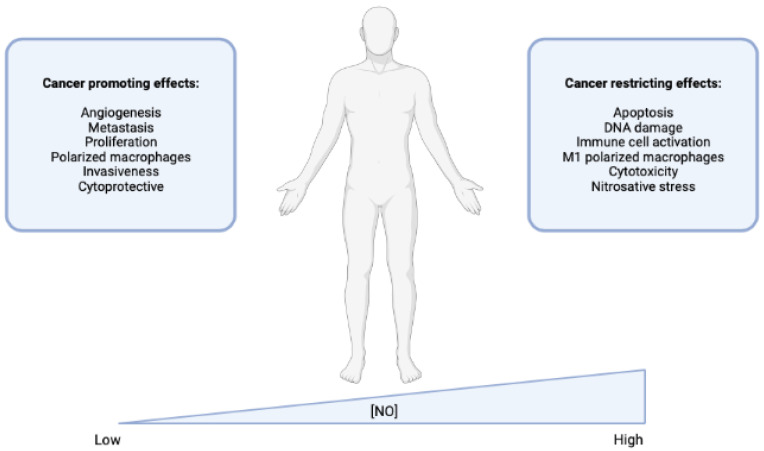
Concentration-dependent effects of NO in cancer. When NO is present in low concentrations, it can improve the molecular processes that maintain normal physiology. However, in already established cancers, low levels of NO may promote cancer progression by enhancing processes such as proliferation and angiogenesis, and the switch to immunologically suppressive immune cell types. In contrast, high levels of NO can induce DNA damage, activate p53, and cause nitrosative stress. While this may promote the development of cancer initially, in already established cancers, high levels of NO can actually activate immunity and improve the effectiveness of chemotherapy. Overall, the effects of NO on cancer depend on the stage of the cancer and the concentration of NO present. While low levels of NO can enhance cancer progression, high levels of NO can induce DNA damage and activate immunity to improve chemotherapeutic efficacy.

NO plays a complex and context-dependent role in cancer, having both pro-tumor and antitumor effects (Figure 4). The effects of NO on cancer cells depend on its concentration and the stage of the cancer. At low concentrations, NO can activate pathways that promote cell growth and inhibit the immune response, leading to the development of cancer. At higher concentrations, NO can induce cell death and inhibit the growth and metastasis of cancer cells [67]. The role of NO in cancer is complex and depends on a variety of factors, including the stage of the cancer and the cellular response to stress. NO can alter the expression of genes involved in DNA repair and tumor suppression, as well as affect processes such as apoptosis and metastasis [68].

Studies have shown that NO can inhibit the growth of various cancer cells, including gastric [69], breast [70], prostate [71], and bladder cancer cells [72], as well as neural precursor cells [73]. GIT-27NO, a novel NO donor, has been shown to inhibit the growth of PC3 and LnCap prostate cancer cells in a concentration-dependent manner when xenografted into nude mice [74]. Saquinavir, a NO-derivative of the HIV protease inhibitor, has also been shown to induce apoptosis and production of proapoptotic BCL-2-interacting mediator of cell death (Bim) in PC3. In vivo studies have shown that Saq-NO inhibited PC3 xenotransplants to a greater extent than the parental compound [75].

One proposed mechanism for the anticancer effects of NO is through the upregulation of a pathway involving BRCA1, Chk1, p53, and p21. This pathway is involved in controlling the cell cycle and can cause cell cycle arrest and cell death in response to DNA damage or other types of cellular stress [76].

Additionally, a study has revealed that NO can increase the sensitivity of cancer cells to radiation, making them more vulnerable to destruction. In cancer radiotherapy, one of the challenges is that hypoxic cells in tumors, which have low levels of oxygen, are not very responsive to radiation. However, NO has been shown to sensitize hypoxic cells to radiation by increasing their oxygen levels through pathways that alter blood flow and oxygen intake by the cells [77]. The combination of NO and ionizing radiation can also induce apoptotic cell death by activating the p53 pathway. In one study, treating colorectal cancer cells with NO donors resulted in a significant increase in the radiosensitivity of the cancer cells [78,79]. Overall, NO has the potential to be a powerful tool in cancer treatment, particularly in combination with radiation therapy. By sensitizing hypoxic cells and inducing apoptotic cell death, NO can improve the effectiveness of cancer treatment and lead to better outcomes for patients.

### 3.4. METAP1 Methionyl Aminopeptidase 1

As said before, tezosentan belongs to the class of organic compounds known as pyridinylpyrimidines that selectively inhibit human methionyl aminopeptidase 1 (*METAP1*) [80,81].

*METAP1* is a protein-coding gene that belongs to the family of metalloproteases. It is involved in the removal of the N-terminal methionine residue from newly synthesized proteins, a process known as N-terminal methionine excision (NME) [82]. *METAP1* is highly conserved across species and is expressed in a wide range of tissues, including the brain, liver, kidney, lung, and heart. It has been implicated in various cellular processes, such as protein synthesis, cell proliferation, and apoptosis [83,84]. Studies have shown that *METAP1* inhibitors have potential therapeutic benefits in the treatment of various diseases, including cancer [83,85] and malaria [86].

Frottin et al., propose that the assessment of *METAP1* levels and redox homeostasis imbalances can be advantageous in creating individualized anticancer tactics. These measurements provide vital information about the role of *METAP1* in cancer and the redox status of cancer cells. By identifying these characteristics in a patient’s cancer cells, healthcare providers can determine the potential effectiveness of *METAP1* inhibitors and redox-targeted therapies in that specific individual. Therefore, *METAP1* levels and redox homeostasis imbalances can serve as innovative tools to develop personalized cancer treatments [83].

## 4. Conclusions

The repurposing of vasodilator drugs such as tezosentan for cancer therapy represents a promising approach to overcome the challenges of current anticancer pharmacological therapies. Tezosentan acts through inhibiting endothelin receptors, which are overexpressed in many types of cancer cells, and has been shown to have potential as a novel anticancer agent. Additionally, tezosentan has shown promising preclinical results in inhibiting cancer cell growth and inducing apoptosis, particularly in tumors with high expression of endothelin receptor type A. Further clinical studies are needed to confirm the efficacy and safety of tezosentan in cancer therapy, but its potential as a repurposed drug for cancer treatment is certainly worth exploring.

One of the aspects that can be developed in the future will be its potential as a new agent to be used in oncology compared to reference drugs if eventually used in combination. In drug combination models, using only repurposed drugs or including reference drugs, can be an excellent way to ensure additive or synergistic effects in a preclinical or even clinical context. Drug combination therapy, in which two or more drugs are used together, aims to increase the chances for better efficacy. Additionally, the efficacy will be observed by decreasing toxicity and reduced development of drug resistance compared to monotherapies. Being a repurposed drug, its clinical safety potential has already been duly proven and this aspect will be a stimulus for further studies involving the drug tezosentan.

## Figures and Tables

**Table 1:** Clinical trials involving ET1 receptors against types of cancer.

Drug	Target	Type of Cancer	Clinical Trial	Status	Identifier Trial Number (https://clinicaltrials.gov) Accessed on 25 April 2023
Atrasentan	ET_A_	Prostate	Phase III	Completed (2021)	NCT00134056
		Prostate	Phase III	Completed (2006)	NCT00036543
		Prostate	Phase III	Completed (2011)	NCT00046943
		Prostate	Phase II	Completed (2010)	NCT00181558
		Prostate	Phase III	Completed (2007)	NCT00036556
		Prostate	Phase II	Completed (2006)	NCT00038662
		Kidney	Phase II	Completed (2010)	NCT00039429
		Ovarian	Phase II	Terminated (2012)	NCT00653328
		Prostate	Phase II/III	Completed (2007)	NCT00127478
		Brain	Phase I	Completed (2009)	NCT00017264
Zibotentan (ZD4054)	ET_A_	Prostate	Phase III	Completed (2016)	NCT00554229
		Prostate	Phase III	Terminated (2012)	NCT00626548
		Prostate	Phase II	Terminated (2019)	NCT01119118
		Prostate	Phase III	Terminated (2012)	NCT00617669
		Prostate	NA	Unknown	NCT01168141
		Breast	Phase II	Withdrawn	NCT01134497
		Lung	Phase II	Completed (2012)	NCT00745875
		Prostate	Phase II	Completed (2013)	NCT00090363
		Prostate	Phase I	Completed (2013)	NCT00314782
		Ovarian	Phase II	Terminated (2012)	NCT00929162
		Prostate	Phase II	Completed (2012)	NCT00055471
		Colorectal	Phase II	Completed (2014)	NCT01205711
BQ788	ET_B_	Melanoma	Phase I	Terminated (2015)	NCT02442466
Bosentan	ET_A_/ET_B_	Pancreatic	Phase I	Recruiting	NCT04158635
		Solid tumor	Phase I	Recruiting	NCT05072106
Macitentan	ET_A_/ET_B_	Glioblastoma	Phase I	Terminated (2018)	NCT02254954
		Glioblastoma	Phase I	Terminated (2016)	NCT01499251

NA—Not applicable.

## Data Availability

Not applicable.
